# Child’s objection to non-beneficial research: capacity and distress based models

**DOI:** 10.1007/s11019-015-9643-8

**Published:** 2015-04-28

**Authors:** Marcin Waligora, Joanna Różyńska, Jan Piasecki

**Affiliations:** Department of Philosophy and Bioethics, Faculty of Health Science, Jagiellonian University, Medical College, Michałowskiego 12, 31-126 Krakow, Poland; Department of Ethics, Institute of Philosophy, University of Warsaw, Krakowskie Przedmieście 3, 00-927 Warsaw, Poland

**Keywords:** Pediatrics, Non-beneficial research, Dissent, Assent, Children, Proxy consent, Incompetent subjects, Vulnerable subjects

## Abstract

A child’s objection, refusal and dissent regarding participation in non-beneficial biomedical research must be respected, even when the parents or legal representatives have given their permission. There is, however, no consensus on the definition and criteria of a meaningful and valid child’s objection. The aim of this article is to clarify this issue. In the first part we describe the problems of a child’s assent in research. In the second part we distinguish and analyze two models of a child’s objection to research: the capacity-based model and the distress-based model. In the last part we present arguments for a broader and unified understanding of a child’s objection within regulations and practices. This will strengthen children’s rights and facilitate the entire process of assessment of research protocols.

## Background

Children’s participation in research that has no potential to produce results of direct benefit to their health (hereafter shortened to “non-beneficial research”) should be allowed by exception only (Berger 2011; Kopelman 2012; Brostrom and Johansson 2014; De Clercq et al. 2014). Due to their limited autonomy and parental dependency, children are considered a vulnerable population that deserves special protection. A total ban on pediatric non-beneficial research would, however, withhold development of pediatric medicine and unjustly deprive children of access to new, safer and more efficient medical interventions (Piasecki et al. 2015). Pediatric research is scientifically needed and morally required by the principle of beneficence, non-maleficence and justice.

Widely accepted research ethics guidelines state that research involving children may be carried out only if the following additional conditions are met: 1. research of comparable effectiveness cannot be performed on competent subjects; 2. research is potentially beneficial for the subject population (e.g. in the same age category or afflicted with the same disease or disorder or with the same condition); 3. research entails only minimal risk or, at least, the risks are minimized; 4. an independent ethical body has examined the scientific and ethical merit of the research; 5. The parents or other legal representatives of the child have given consent; 6. the child’s opinion has been taken into account (WMA 2013; CIOMS 2002; CoE 1997, 2005; EC 2001). This last requirement usually entails an obligation to obtain the minor’s agreement—often known as assent—and an obligation to respect his/her objection or dissent.

In the first part of this article, we describe the concept of child’s assent in research. In the second part, we distinguish and analyze two models of a child’s objection to research: the capacity-based model and the distress-based model. In the third part, we present arguments for a broader and unified understanding of a child’s objection, also encompassing a distress-based model. The terms “minor” and “child” are used in the paper interchangeably, indicating individuals who have not reached the legal age of adulthood and have no legal competence to give informed consent.

## Child’s assent for research

All international guidelines recognize that participation in research should be voluntary and guided by the principle of respect for autonomy. In the case of research on adults, this principle finds its expression in the legal and ethical obligation to seek subjects’ free and informed consent. With pediatric research, the principle gives rise to an obligation to gain parental or proxy informed consent and involve minors in the research participation decision-making process. Children, who have no legal competence to give valid consent for their participation in research should be informed to the extent that their understanding permits about the research proposal and asked for their assent to participate. Thus, the permission of a child’s parents or other legal representatives should be supplemented by the child’s agreement.

Although it is internationally recognized that investigators are obliged to seek a decisionally capacitated child’s assent, there are considerable differences between the guidelines on the definition and significance of the assent. Some, for instance the WMA Declaration of Helsinki, and the Council of Europe Oviedo Convention with the Additional Protocol to the Convention concerning biomedical research, do not say anything on how the assent should be interpreted. Other research guidelines provide more details on assent, but there is no consensus on what constitutes assent, what intellectual and decisional capacities are necessary to express it, and, consequently, which children are capable of giving a *meaningful* assent to their participation in research (WMA 2013; CoE 1997, 2005). Depending on the understanding of the minor’s assent that is adopted, different decision-making capacity standards have been proposed in the regulatory documents and literature. For instance, the European Commission Ad Hoc Group for the Development of Implementing Guidelines for Directive 2001/20/EC—“Ethical Considerations for Clinical Trials Performed on Children” suggest an age threshold for assent in pediatric research (Ad Hoc Group 2006). Although the Clinical Trials Directive does not explicitly mention the notion of assent, the Group recommends that in addition to the informed consent of the legal representative, the assent of the child of “school age” (about 6 or 7 years old) should be sought. The Group defines “assent” as “the expression of the minor’s will to participate in a clinical trial” (sec. 5 and 7) (Ad Hoc Group 2006). It further notes that the capacity to assent, i.e. to make a voluntary and informed decision, evolves with age, but also depends on other factors such as the developmental stage, maturity and previous experience of life and illness. Discussing the minor’s capacity to assent, the Group focuses mainly on his/her ability to understand the research protocol and to make independent decisions (sec. 7.1.2) (Ad Hoc Group 2006).

Some ethicists and researchers define the capacity to assent in a more complex and stringent way, almost equating it with the capacity to give informed consent. The CIOMS Guidelines seem to assume such an understanding. In the commentary on Guideline 14, assent is defined as a “knowing agreement” of the child obtained “after the child has been informed to the extent that the child’s maturity and intelligence permit”. The legally incompetent child is able to knowingly agree to serve as a research subject if the child “can understand the implications of informed consent and go through the necessary procedures”. The Commentary further states that “it may be assumed that children over the age of 12 or 13 years are usually capable of understanding what is necessary to give adequately informed consent”, and therefore, their assent (if not consent) must be obtained (CIOMS 2002).

The above overview shows that there is no commonly accepted standard for what age or level of intellectual and emotional capacity makes the child able to give a meaningful assent for research. However, it is important to note that there is a growing consensus that context-dependent methods of assessing an individual capacity to assent/consent could be employed (Hein et al. 2012, 2014). A child’s capacity to be meaningfully involved in the research participation decision-making process does not depend only on his/her age status, but also on previous experiences, family relationships, in particular parents’ willingness to offer autonomy to the child and solicit his/her views, the quality of communication between the investigator, the child and parents, and the nature of the decision to be made (Alderson 2007; Joffe 2003; Kon 2006; Miller et al. 2004; Cheah and Parker 2014; Leibson and Koren 2014). This approach is sometimes called “personalized assent”, and we discussed it elsewhere (Giesbertz et al. 2014a, b; Waligora 2014; Giesbertz et al. 2014b; Waligora et al. 2014).

## Child’s objection—capacity or distress-based notion?

As discussed, international guidelines define a child’s assent to research in a different and often unclear manner. But they are even vaguer when it comes to a child’s objection to participation. It is internationally recognized that—in principle—the child’s objections must be respected by researchers, even when the parents or legal representatives have given permission. There is, however, no consensus on definition and criteria of a meaningful and valid child’s objection. Two models can be found in international standards: (1) the capacity-based model and (2) the distress-based model.

The capacity-based model is rooted in the principle of respect for (developing) autonomy. It can be found in the current version of the Declaration of Helsinki and in the EU Clinical Trials Directive. Paragraph 29 of the Declaration reads: “When a potential research subject who is deemed incapable of giving informed consent is able to give assent to decisions about participation in research, the physician must seek that assent in addition to the consent of the legally authorized representative. The potential subject’s dissent should be respected”. Taken literally, the quoted paragraph seems to imply that the ethical validity of a child’s dissent depends on his/her decisional capacity to give assent. An investigator is obliged to respect a child’s dissent only if the child has the understanding and decisional capacities required for giving a meaningful assent. In other words, dissent stands for negative assent.

The capacity-based model is also adopted by the EU Clinical Trials Directive. Article 4 of the Directive states that, in addition to other relevant restrictions, a clinical trial on minors may be undertaken only if: “(b) the minor has received information according to its capacity of understanding, from staff with experience with minors, regarding the trial, the risks and the benefits; (c) the explicit wish of a minor who is capable of forming an opinion and assessing this information to refuse participation or to be withdrawn from the clinical trial at any time is considered by the investigator or where appropriate the principal investigator”. Similarly to the Declaration of Helsinki, the Directive assumes that to be able to express a meaningful objection a child must have certain intellectual and decisional capacities. Contrary to the Declaration, the Directive explicitly requires investigators only to take the minor’s objection under consideration. Furthermore, the new European Union Regulation repealing the Directive offers a similar approach on this matter.

The second child’s objection model—the distress-based model—may be found in the CIOMS Guidelines as well as in the Oviedo Convention and the Additional Protocol to the Convention concerning Biomedical Research. Guideline 14 of the CIOMS Guidelines states that, as a rule, an investigator should respect a child’s refusal to participate or continue in the research. Only when research shows the promise of therapeutic benefit for a child and there is no alternative therapy may the child’s refusal to participate or continue in the research be overruled by parents. The commentary on the Guideline further explains that even children who “are too immature to be able to give knowing agreement, or assent, may be able to register a ‘deliberate objection’, an expression of disapproval or refusal of a proposed procedure”. However, the deliberate objection must be distinguished from “the behavior of an infant, who is likely to cry or withdraw in response to almost any stimulus”. This means that not all signs of distress should lead to immediate withdrawal of a child from research. Some negative reactions of a child might be caused by non-research-related circumstances or non-essential aspects of the research (Wendler and Shah 2003). If possible, these circumstances or research aspects should be eliminated, and the participation might be continued. Some reactions might be caused by sudden emotions, which can change fast (Joffe 2003). Therefore, the child’s behavior must be interpreted appropriately. It is generally agreed that this is the role of researchers and, above all, parents (John et al. 2007). In most cases, parents are in the best position to interpret their child’s behaviors. Moreover, society respects parental decisions about many aspects of their children’s lives. Thus the important parental role in interpreting signs of a child’s distress is consistent with general social rules.

Moreover, the Oviedo Convention and the Additional Protocol concerning Biomedical Research stipulate that research on a child may be carried out only if the child concerned does not object (Article 17(1)v, and Article 15(1)v respectively). The Explanatory Report to the Protocol (sec. 106) explains that the objections may be expressed by non-verbal means. In interpreting the wishes of children, their age and maturity should be taken into account, and—in the case of those unable to express themselves—the opinion of the parents or other caregivers.

Both the CIOMS Guidelines and the Council of Europe regulations indicate that a child must possess some intellectual capacities to express a valid objection. However, the distress-based model does not require an objecting child to have capacities as developed and sophisticated as the one required for giving a meaningful assent. The child must be able to form and communicate a preference, even non-verbally. (Joffe 2003; Spriggs 2010). In the case of non-beneficial research, the sole fact that the child perceives the investigational procedure as distressing, unpleasant or burdensome seems to be sufficient to withdraw him/her from the research program. Thus, the principle of non-maleficence may be considered the main moral justification for the distress-based model in the context of research (Wendler 2008; Wendler and Shah 2003).

## Argument for respecting distress-based objection

According to the capacity-based model, only the objection of a decision-making-capacitated child is meaningful and has a moral value. Therefore, in this model, when a child is considered unable to give meaningful objection, the child’s opposition does not have strong moral significance and does not have to be taken into serious consideration by the researchers or parents. It is our view that the regulatory requirement to respect a child’s objection to research should be interpreted as also encompassing the distress-based objection.

We argue that children’s participation in research that has no potential to produce results of direct benefit to their health is a supererogatory act. If the act is supererogatory, then the refusal to participate in it does not need to be rational, informed and justified.

The current paradigm of research ethics and law is founded on the assumption of the supererogatory nature of research. Commentators agree that participation in biomedical research is a moral good. However, this is only one good among many. This moral good provides a reason to participate in research. Since one does not have a duty to do all possible moral good, one also is not obliged to choose this particular one, participation in research (Brassington 2011). The moral duty to participate in biomedical research can appear only in some rare, emergency situations (Shapshay and Pimple 2007). Therefore, the following analysis rejects the obligation model in research ethics and refers to the notion of supererogation. The obligation model is alien to the dominant regulatory framework in research that stresses the voluntariness of the subject’s decision to participate in a study and his/her right to withdraw consent at any stage, without any justification.

Accepting David Heyd’s model of supererogation, we state that participation in non-beneficial research might be described as a supererogatory act. The burden of proof that it is otherwise remains on the opponents of this view—they have to give an argument showing that a given act is a moral obligation. However, it seems that to date, all arguments supporting the obligatory nature of participation in research remain unconvincing (Brassington 2011, 2013; Shapshay and Pimple 2007; Lyons 2011; Rennie 2011). An act is supererogatory, according to Heyds, when “(1) It is neither obligatory nor forbidden. (2) Its omission is not wrong, and does not deserve sanction or criticism—either formal or informal. (3) It is morally good, both by virtue of its (intended) consequences and by virtue of its intrinsic value (being beyond duty). (4) It is done voluntarily for the sake of someone else’s good, and is thus meritorious”(Heyd 2012).

Participation in non-beneficial research seems to meet all these criteria. For instance, donation of blood samples or nasal swabs for research by healthy volunteers is not forbidden, but also not obligatory. Most members of the world human population will never participate in such a procedure; many decline participation when asked. The ethics and law regulating biomedical research support the view that this omission is not wrong, and does not deserve sanction. Any type of formal or informal criticism toward refusal to participate could be classified as an undue inducement. On the other hand, participation in this kind of minimal risk research, which potentially helps in developing new drugs or procedures (morally good intentions), is good in a moral sense. This is a meritorious act: it could be done voluntarily for the good of future patients.

The supererogatory nature of non-beneficial research provides justification for the voluntary character of participation in such research. Informed consent is a necessary condition of participation of a capacitated individual. However, while consent to participate in research must be informed, lack of consent or refusal does not have to be. Lack of consent can be based only on non-informed distress. If a researcher invites a 31-year-old competent woman to take part in non-beneficial research based on a blood sample or nasal swab donation, she can refuse before being informed about the nature of the research. No one can force her to become familiar with the information sheet or to discuss the research proposal with an investigator. Her ignorant refusal to participate in this supererogatory procedure must be accepted because she has no obligation to participate in this research, and no obligation to listen and talk to the researcher. She is also not obliged to base her own refusal on knowledge about this research. Since the current approach to research ethics accepts ignorant refusals of adults, we do not find any arguments for not accepting ignorant, even only distressed-based refusals of incompetent minors. In both cases, the subjects’ objections are just an expression of their preferences. Both groups know almost nothing about the proposed research, but they decline to participate, and their decisions should be respected.

Furthermore, participation in the decision-making process of significant events in a child’s life has an important role in the child’s development (Giesbertz et al. 2014a, b; Sibley et al. 2012). Giving the child an opportunity to object to participation or continuation in research may be considered a lesson in making an autonomous choice. Engaging even incapacitated children can have an immense impact on their developing autonomy and can strengthen their abilities to make responsible choices and behave autonomously in the health context (Alderson et al. 2006).

Both medicine and research are a joint venture; thus cooperation with a young patient and research subject is a necessary condition. Giving information and making a real agreement that objection would be respected is not only beneficial from a psychological point of view, but also helps to preserve trust in healthcare professionals, physicians, and parents (Spriggs 2010). In a healthcare setting, cooperation even with a very young child is possible. Therefore, the respect for distressed-based objections is one of the elements building a relationship between the researcher, child and parents that is professional and founded on trust. The quality of these relations may have a strong impact on the child’s willingness to cooperate with the physician and participate in future research.

## Summary

Despite the fact that many detailed regulations exist, children’s participation in biomedical research still raises many questions. The lack of clarity of definitions, absence of detailed instructions, varying approaches by international standards and diverse practices mean that children’s decision-making status is insufficiently clear. The vaguest area is the issue of a child’s objection to research. This article presents arguments for a broader and unified understanding of a child’s objection within regulations and practices. Researchers and parents should respect not only capacity-based, but also distressed-based child’s objections. This will strengthen even very young children’s rights and facilitate the entire process of assessment of research protocols.
